# Max Bielschowsky (1869–1940)

**DOI:** 10.1007/s00415-014-7544-z

**Published:** 2014-10-28

**Authors:** F. W. Stahnisch

**Affiliations:** Departments of Community Health Sciences and History, Hotchkiss Brain Institute and O’Brian Institute for Public Health, University of Calgary, 3280 Hospital Drive N.W., Calgary, AB T2N 4Z6 Canada

**Keywords:** Berlin, Clinical neurology, History of neurology, Neurohistology, Max Bielschowsky, Twentieth century, London

## Abstract

Berlin neurologist and neurohistologist Max Bielschowsky counts among the most innovative microanatomical researchers at the beginning of the twentieth century. Although being quite underrated in the history of neurology today, Bielschowsky contributed substantially to the understanding of neurohereditary pathologies, such as Alzheimer’s disease, Parkinsonism, and Huntington’s chorea, as well as the assessment of structural changes in several movement disorders. Working with other leading research neurologists, such as Oskar and Cecile Vogt or Korbinian Brodmann at the newly founded Kaiser Wilhelm Institute for Brain Research in Berlin-Buch, he also pioneered neurohistological work on de- and regeneration processes in the Central Nervous System along with new morphological definitions of “nervous trauma.”


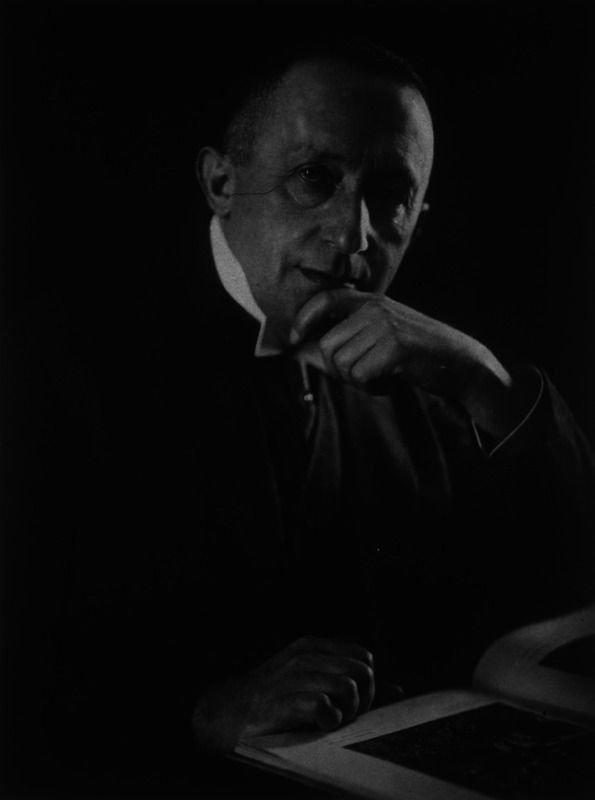
Max Bielschowsky (1869–1940) was born on 19 February, 1869 in Breslau (Silesia) as the son of Natalie Lion (1839–1918) and businessman Eduard Bielschowsky (1840–1910). Max Bielschowsky was also a cousin of the ophthalmologist and strabismus researcher Alfred Bielschowsky (1871–1940)—who was himself the offspring of a notable family of academics in the nearby Silesian city of Namslau—after whom the head tilt test for superior oblique muscle palsy is named. Following Max Bielschowsky’s MD graduation at the University of Munich in 1893, he pursued postgraduate training in Frankfurt am Main and Berlin [6]. While working with neuroanatomist Ludwig Edinger (1855–1918) at the Senckenberg Institute, he was introduced to cutting-edge microscopic research techniques. From 1896 to 1904, Bielschowsky joined one of the most prominent research neurologists, Emanuel Mendel (1839–1907), who interested him in the clinical applications of neuropathological work [4].

Hereafter, Bielschowsky assumed the position of a research associate of Oscar Vogt (1870–1959) [8], the neuropathologist and inaugural director of the Berlin Kaiser Wilhelm Institute for Brain Research, but in 1933, Bielschowsky lost his academic headship of the neuropathology department due to the inauguration of the Nazi’s anti-Semitic laws. He remained in Berlin as a physician in private practice for another 2 years, but left Germany in 1935, and joined the University of Utrecht in the Netherlands. When the Second World War broke out, he fled to Spain to work at the Cajal Institute for Brain Research in Madrid, before finally emigrating to the UK, where he died on 15 August, 1940 in the Greater London area at 71 years of age [10].

Bielschowsky made major contributions to the understanding of common pathologies of the central nervous system, such as Alzheimer’s disease, Parkinsonism, Huntington’s chorea, and degenerative movement disorders. Through the development of his research programme, clinical symptoms became increasingly related back to the pathological findings in post-mortem analyses along with the general morphological qualities of disease—pertinent also in the work of prominent contemporary neurologists and psychiatrists such as Otto Binswanger (1852–1929) in Jena, Oswald Bumke (1877–1950) in Leipzig and Munich, as well as Walther Spielmeyer (1879–1935) in Munich. The scientific discourse about what defined de- and regeneration phenomena now essentially became deferred to a new privileged place of medical knowledge production—the neuromorphological laboratory:

“Since we have come to know that the parenchyma of the nervous central organs is made up from ganglia cells and nerve fibres, we also have created the problem, in which ways these cells and fibres are interrelated with each other” [1].

Bielschowsky’s further work on Traumatic Regeneration Processes in the Central Nerve Fibres (1909) [2] emerged from well-defined and encapsulated research in the traditional departments of anatomy, biology, and pathology. Psychiatrists, neurologists, and clinical pathologists, however, now all began to use the new staining methods, even though they worked in quite different experimental settings and, of course, with different research objects according to the respective laboratory traditions. Yet the common denominator of all these endeavours was their interest in analysing clinical phenomena in human patients in what they considered as adequate laboratory models [9].

Bielschowsky worked closely together with other scientists in Oskar and Cécile Vogt’s (1875–1962) “neurological laboratory” in downtown Berlin—for example with Korbinian Brodmann (1868–1918), who pursued there his influential research on the neurophysiological architecture of the human cortex—as was later reflected in Bielschowsky’s handbook of human microscopic anatomy (1928). Following the last years of the Great War, with its horrendous amounts of casualties, Bielschowsky progressively collaborated with the neurosurgeons Maksymilian Rose (1883–1937) and Ernst Unger (1875–1938) on problems of nerve trauma [3]. The laboratory pursuit of “de- and regeneration” in German brain science had been significantly influenced by the succession of various societal contexts from the late Wilhelminian epoch to the interwar period. Instructive cases are, for example, the Berlin neurologist Hermann Oppenheim (1858–1919) and later Heidelberg psychiatrist Victor von Weizsaecker (1886–1957). For Oppenheim and von Weizsaecker alike, the notion of “trauma” became integrated in an anthropologico-medical approach of the “healing of the social body” [7], whereas for Bielschowsky it rose to a predominantly neuromorphological and likewise genetic meaning. The experience of trauma was reinterpreted by Bielschowsky as an inherited disposition in what was seen as “degenerate patients,” while morphological changes of the brain could be brought about through physical as well as psychological wounds—as was described in the “Investigations of the Processes of the Staining of the Connective Tissue following the Method of Bielschowsky-Maresch” (1924) by his former pupil Dr. Hans Loewenstaedt (b. 1882) in Wiesbaden [5].

It was quite clear to Bielschowsky that this research would have further implications for questions of nervous de- and regeneration as well. As a former military physician, Captain Max Bielschowsky had been convinced about the existence of positive nervous regenerative phenomena, but later as head of the neuropathology department at the Kaiser Wilhelm Institute for Brain Research, he became less optimistic as to the functional potential of regenerative phenomena in the central nervous system. With his laboratory investigations, as for example described in his textbook entries on “Nervous Tissue” in the Handbook of Human Microscopic Anatomy (1928)—edited by Zurich anatomist Wilhelm von Moellendorff (1887–1944)—now placed in the context of Weimar degeneration discourses of the 1920s, Bielschowsky reinterpreted axonal growth phenomena, glia cell invasion, and continuous repair mechanisms largely as forms of abortive nerve growth processes.

Where previously the views of biological psychiatrists, such as Emil Kraepelin (1856–1926) and Oswald Bumke in Munich had limited the notion of “trauma” to psychological factors, and Sigmund Freud (1856–1939) in Vienna as well as other psychoanalysts had cemented the aetiological interpretation of “nervous trauma” in their psychopathology, Bielschowsky and several Weimar neurohistologists—including Walther Spielmeyer (1879–1935) in Munich, Karl Stern (1906–1975) in Frankfurt, and Hans Altenburger (1902–1938) in Breslau—sought to provide microscopical analyses of such “degenerative occurrences:”

“And now the silver-image! When observing the preparation with the unaided eye, one is quite astonished not to find a distinct border between the normal and the sclerotic tissue. […] The diameter has a homogenous grey or black appearance; under the microscope one can see abundant nerve fibres in the sclerotic area which are as dense as those in the areas filled with nerves which have myelin sheets [*markhaltige Nervenzellen *...].  Eventually, a long-standing clinical desideratum could thus be solved” [1].

Bielschowsky had provided an important foundation for this new neurohistological research direction with the invention of his derivative silver staining technique (1902/1904)—which still bears his eponym—representing catalytic “nuclei” particularly in degenerative neurons with, for example, the neurofibrillary tangles and senile plaques in Alzheimer’s disease. During the Weimar period, numerous publications from Bielschowsky’s neuropathology department, devoted to the contemporary problems of neuronal de- and regeneration, nerve trauma, and the treatment of nerve lesions, appeared in the leading medical journals. They underlined Bielschowsky’s enormous anatomical productivity and contributions to the ongoing discourses on “nervous trauma” at the time. However, his personal wish to make inroads into the clinical discussions and transform pathophysiological views on the psychological and somatic influences on the de- and regenerative capacities of the central nervous system remained rather limited.

## References

[1] Bielschowsky M (1904) Die Silberimpraegnation der Neurofibrillen. J Psych Neurol;3:169–189 [esp 182 and 186f; all emphases as well as translation by FWS].

[2] Bielschowsky M (1909). Ueber Regenerationserscheinungen an zentralen Nervenfasern. J Psychol Neurol.

[3] Bielschowsky M, Unger E (1917). Die Ueberbrueckung grosser Nervenluecken. J Psychol Neurol.

[4] Holdorff B (1999). Hermann Oppenheim (1858–1919) and Max Lewandowsky (1876–1918) – ein Vergleich. Schr Deutsch Ges Gesch Nervenhlk.

[5] Loewenstaedt H (1924). Untersuchungen ueber die Vorgaenge bei der Bindegewebsversilberung nach Bielschowsky-Maresch und ueber die Konstitution der ‘Gitterfasern’. Z f ges exp Med.

[6] Ostertag B (1963) Max Bielschowsky (1869–1940). In: Kolle K (ed) Grosse Nervenaerzte, Stuttgart, Thieme, Vol 3, pp. 3–8.

[7] Radkau J (1998). Das Zeitalter der Nervositaet. Deutschland zwischen Bismarck und Hitler.

[8] Stahnisch FW, Helmchen H (2008). Psychiatrie und Hirnforschung: Zu den interstitiellen Uebergaengen des staedtischen Wissenschaftsraums im Labor der Berliner Metropole – Oskar und Cécile Vogt, Korbinian Brodmann, Kurt Goldstein. Psychiater und Zeitgeist. Zur Geschichte der Psychiatrie in Berlin.

[9] Stahnisch FW (2009). Transforming the Lab: Technological and Societal Concerns in the Pursuit of De- and Regeneration in the German Morphological Neurosciences, 1910–1930. Med Stud.

[10] Will M (2000) Max Bielschowsky – Eine Bioergographie, Muenster, Diss Med, pp. 2–18 and 51–53.

